# News and Coming Events

**Published:** 1909-01-23

**Authors:** 


### News and Coming Events.

The Countess Bathurst has become a Vice-Patron of the Chelsea Hospital for Women.

Sir Frederick Treves, Bart., will deliver a lecture on Radium in Surgery at the London Hospital Medical College, on Tuesday, January 26, at 3 p.m.

The German Urological Society will hold its second Congress at Berlin, April 18 to 22, 1909. Among the subjects to be discussed are suppurative non-tuberculous affections of the kidneys, and tumours of the bladder. An exhibition will be held in connection with the Congress. All communications should be addressed to Sanitätsrat Dr. Wossidlo, Victoriastrasse 19, Berlin, W.

At a meeting of the Royal Statistical Society held on Tuesday, January 19, at 5 p.m., at the Society's Rooms, 9 Adelphi Terrace, Strand, London, W.C., a paper was read, entitled "The Cost, Conditions, and Results of Hospital Relief in London" (Howard Medal Prize Essay), by Mr. Percy E. Braun, B.Sc. (Econ.). The paper dealt with the following points: Hospitals under trustee management; percentage of cost of management to total ordinary expenditure; hospital statistics; application of the statistics to the accounts; cost of provisions per bed occupied; domestic charges per bed occupied; hospital income; patients' payments and classes of beneficiaries.

The work of the Medical Graduates' College and Polyclinic, 22 Chenies Street, London, W.C., was resumed on January 18, when the first clinic of the present session was held at 4 p.m.

The Festival Dinner of the Hampstead General Hospital (with which is amalgamated the North West London Hospital) will be held at the Trocadero Restaurant, Shaftesbury Avenue, W.C., on Tuesday, February 16, at 6.30 p.m. for 7 p.m., under the chairmanship of Samuel Figgis, Esq.

Dr. Robert Jones has been elected to succeed Dr. T. Claye Shaw as lecturer on mental diseases at St. Bartholomew's Hospital and he has therefore resigned a similar post at the Westminster Hospital Medical School, which he has held for some years.

The Duchess of Albany presided at a meeting of the Executive Committee of the Jubilee Fund of the National Hospital for the Paralysed and Epileptic, Queen Square, Bloomsbury, on January 18. It was announced that over £11,000 had been collected and promised towards the £50,000 for which the Duchess is appealing, including a donation of £1,000 from Lord Strathcona, the president of the hospital. It was reported that the Lord Mayor has promised to preside at a dinner in aid of the fund at the Mansion House on June 10 next.

Dr. T. M. Allison, M.D., has been appointed honorary assistant physician to the Royal Victoria Infirmary, Newcastle-on-Tyne.

Mr. Phineas Abraham, M.D., F.R.C.S.I., has resigned his post of surgeon to the Hospital for Diseases of the Skin, Stamford Street, Blackfriars.

The Morison lectures of the Royal College of Physicians of Edinburgh will this year be delivered in the College Hall, 9 Queen Street, by Dr. F. W. Mott, F.R.S., at 5 o'clock p.m., on Monday, January 25, Wednesday, January 27, and Friday, January 29, the subject being "Syphilis of the Nervous System in the Light of Modern Research."

The Council of the Sheffield University on January 18 considered a report prepared by the Vice-Chancellor on the application of the increased income which will accrue to the University under the will of the late Dr. Hunter, of Bath, who left the institution £15,000. In accordance with the terms of the late Dr. Hunter's will the name of his father, Joseph Hunter, has to be associated with the fund. The Council referred the report to a joint committee of the Council and Senate, to consider how far the establishment of a chair in some branch of economic science, and the provision of instruction for theological students, can be met by the resources available. This committee is also to consider how the name of Joseph Hunter may be associated with the faculty of medicine.

Lord Derby, the President, on Jan. 16, laid the foundation-stone of a new Liverpool Dental Hospital, which is to be erected on the site near the University and the Royal Infirmary. The building, together with its equipment, is to cost £10,000, and of this sum £7,500 has already been promised or subscribed, £1,000 having been promised by Sir William P. Hartley, £100 contributed by the hospital staff, £800 by Mrs. and Miss Holt, and £500 by Sir John Brunner, M.P. The present hospital was founded in 1876. Over 27,000 patients were attended to last year, and upwards of 16,000 teeth saved, this being the first time in the history of the institution that more teeth were saved than had to be extracted. Lord Derby on this occasion made his first public appearance in Liverpool since his accession to the title.

At the Central Criminal Court on January 14, before the Recorder, Jane Emily Inglis pleaded guilty to an indictment charging her with procuring a certificate of admission to the Midwives' Roll by a false certificate. Mr. Bodkin, for the prosecution, said this was the first case under the Midwives Act. The prisoner practised as a nurse at Bradford and she induced a local medical man to give her a certificate which stated that she was trustworthy and sober, and as a result she was placed on the Roll of Midwives. Afterwards it was discovered that in 1892 the prisoner had been tried for murder and was sentenced to penal servitude for life for performing an illegal operation which ended fatally. This sentence was commuted to three years' penal servitude. After her release she was again charged with a similar offence, but was acquitted. The local doctor, when communicated with, denied all knowledge of these cases, and explained that the prisoner was introduced to him by his assistant, now dead, and his certificate was fully justified by what he knew of her since. Mr. Curtis Bennett addressed the Court on behalf of the prisoner. The Recorder said the prosecution was a very proper one in the public interest, but decided not to deal with the prisoner until next sessions.

H.R.H. the Princess of Wales has intimated her intention of visiting the Great Northern Central Hospital on Monday, February 22, in the afternoon, to open the new ward for children provided through funds collected by the Ladies' Association, of which her Royal Highness is the President.

Among the latest contributions received at the Bank of England for King Edward's Hospital Fund for London are the following annual subscriptions: Mr. William Waldorf Astor, £1,000; Messrs. C. J. Hambro and Son, £250; Prudential Assurance Co., Limited, £250; Messrs. Wernher, Beit and Co., £250; Mrs. Walter H. Burns, £200.

Dr. J. J. Charles, M.D., D.Sc., F.R.S.E., formerly professor of anatomy and physiology in Cork University College, has presented an engraved die, and a sum of money sufficient to provide an annual gold medal, to be given alternately in the subjects of which Dr. Charles was professor, but which are now under the teaching of two professors. The governing body have accepted this offer, and ordered that the medal shall be called the "Charles Medal."

At the last quarterly meeting of the Council of the Royal College of Surgeons on January 14, Mr. Henry Morris, president, being in the chair, a formula for a new by-law relating to the admission of women to the examinations for the diplomas of Fellowship and Membership and of the License in Dental Surgery was submitted and was referred to a committee to take into consideration and report thereon to the next meeting of the Council. A vote of thanks was accorded to Mr. E. L. Gruning for his gift of skulls from Cook Islands, of pathological as well as of anthropological interest. Mr. Henry T. Butlin, F.R.C.S., was re-elected a member of the Court of Governors of the University of Birmingham.

We regret to announce the death of Dr. John Evan Spicer, medical registrar at the London Hospital, which occurred on Saturday, January 16, on the Alps, near Leuzer-Leide. Dr. Spicer was carried down a deep gorge by an avalanche, whilst upon a ski tour with his younger brother, who was subsequently rescued in an exhausted condition by a search party. Dr. Spicer received his medical education at Cambridge and the London Hospital, and his untimely death at the age of thirty-two cuts off a most promising career. He qualified in 1903, and held various resident posts before being appointed to a medical registrarship at the London Hospital. He took the M.D. degree at Cambridge University in 1908, and had already contributed several papers to medical and scientific journals.

